# Supplementary material for: Body image and appearance distress among military veterans and civilians with an injury-related visible difference: A comparison study
Source: PLoS One. 2025 Feb 7;20(2):e0305022. doi: 10.1371/journal.pone.0305022 (PMC11805358; doi:10.1371/journal.pone.0305022)
Supplement: S1 Table — (DOCX) [file pone.0305022.s001.docx]

# Body image and appearance distress among military Veterans and civilians with an injury-related visible difference: A comparison study

Keeling, M., Harcourt, D., White, P., Evans, S., Williams, V.S., Kiff, J., and Williamson, H.

Submitted to PLOS ONE.

# Supplementary Information S1

Regression analyses based on multiple imputation (M = 100)

Table S1. Pooled Multiple Imputation Regression Models for Appearance Outcomes

|  | BESAA-AE | | | | | | |
| --- | --- | --- | --- | --- | --- | --- | --- |
|  | Veteran | | |  | Civilian | | |
| Measure | Beta | t | p |  | Beta | t | p |
|  |  |  |  |  |  |  |  |
| BIAAQ | .567 | 5.753 | <.001 |  | .439 | 5.893 | <.001 |
| BICSI | -.091 | -1.253 | .210 |  | -.216 | -3.758 | <.001 |
| PSQ | -.022 | -0.295 | .768 |  | .032 | 0.528 | .698 |
| SCS-SF | .004 | 0.036 | .972 |  | .180 | 2.464 | .014 |
| EMAS | .251 | 3.573 | <.001 |  | .118 | 2.050 | .040 |
| LOT-R | .050 | 0.490 | .624 |  | .124 | 1.781 | .075 |
| MSPSS | .006 | 0.078 | .938 |  | -.028 | -0.515 | .607 |
| PTSD | .006 | 0.069 | .945 |  | .055 | 0.778 | .437 |
|  |  |  |  |  |  |  |  |
|  | BILEQ | | | | | | |
|  | Veteran | | |  | Civilian | | |
|  | Beta | t | p |  | Beta | t | p |
| BIAAQ | .563 | 6.309 | <.001 |  | .667 | 8.955 | <.001 |
| BICSI | -.010 | -0.155 | .877 |  | .145 | 2.457 | .014 |
| PSQ | -.048 | -0.716 | .474 |  | -.171 | -2.834 | .005 |
| Self-compassion | -.038 | 0.405 | .686 |  | -.164 | -2.255 | .024 |
| EMAS | .123 | 1.923 | .054 |  | .161 | 2.852 | .004 |
| LOT-R | .057 | 0.620 | .535 |  | .090 | 1.329 | .184 |
| MSPSS | .080 | 1.214 | .225 |  | .004 | 0.086 | .932 |
| PTSD | -.197 | -2.242 | .025 |  | -.045 | -0.661 | .508 |
|  | FNAE | | | | | | |
|  | Veteran | | |  | Civilian | | |
|  | Beta | t | p |  | Beta | t | p |
| BIAAQ | -.455 | -5.535 | <.001 |  | -.281 | -4.908 | <.001 |
| BICSI | .322 | 5.501 | <.001 |  | .497 | 11.214 | <.001 |
| PSQ | .020 | 0.323 | .747 |  | .031 | 0.664 | .507 |
| SCS-SF | -.135 | -1.567 | .117 |  | -.174 | -3.100 | .002 |
| EMAS | -.015 | -0.255 | .799 |  | -.017 | -0.380 | .704 |
| LOT-R | .047 | 0.568 | .570 |  | -.084 | -1.614 | .107 |
| MSPSS | -.002 | -0.040 | .968 |  | -.030 | -0.744 | .457 |
| PTSD | .135 | 1.746 | .081 |  | .002 | 0.043 | .966 |
| BESAA-AE: Body Esteem – Appearance Sub-scale; BILEQ: Body Image Life Disengagement; FNAES: Fear of Negative Appearance Evaluation. BIAAQ: Body Image Acceptance and Action (Body Image Psychological Flexibility; BICSI-AF: Body Image Coping Strategies – Appearance Fixing; PSQ: Perceived Stigma; SCS-SF: Self-Compassion; EMAS: Engagement in Meaningful Activities; LOT-R: Optimism; MSPPS: Multidimensional Perceived Social Support; ITQ: International Trauma Questionnaire (PTSD). | | | | | | | |
