# Supplementary material for: Body image and appearance distress among military veterans and civilians with an injury-related visible difference: A comparison study
Source: PLoS One. 2025 Feb 7;20(2):e0305022. doi: 10.1371/journal.pone.0305022 (PMC11805358; doi:10.1371/journal.pone.0305022)
Supplement: S2 Table — (DOCX) [file pone.0305022.s002.docx]

# Body image and appearance distress among military Veterans and civilians with an injury-related visible difference: A comparison study

Keeling, M., Harcourt, D., White, P., Evans, S., Williams, V.S., Kiff, J., and Williamson, H.

Submitted to PLOS ONE.

**Supplementary Information S2**

Regression analyses based on multiple imputation (M = 100)

Table S2. Pooled Multiple Imputation Regression Models for PTSD and Social Anxiety

|  | PHQ-9 | | | | | | |
| --- | --- | --- | --- | --- | --- | --- | --- |
|  | Veteran | | |  | Civilian | | |
| Measure | Beta | t | p |  | Beta | t | p |
|  |  |  |  |  |  |  |  |
| BIAAQ | -.059 | -0.666 | .506 |  | -.075 | -0.904 | .366 |
| BICSI | -.110 | -1.692 | .091 |  | -.065 | -1.067 | .286 |
| PSS | -.021 | -0.318 | .751 |  | -.002 | -0.034 | .973 |
| SCS-SF | -.256 | -2.744 | .006 |  | -.356 | -4.719 | <.001 |
| EMAS | -.104 | -1.645 | .100 |  | -.158 | -2.655 | .008 |
| LOT-R | -.147 | -1.668 | .095 |  | -.060 | -0.861 | .389 |
| MSPSS | -.074 | -1.180 | .238 |  | -.069 | -1.294 | .196 |
| PTSD | .444 | 5.339 | <.001 |  | .358 | 5.074 | <.001 |
|  |  |  |  |  |  |  |  |
|  | SAS-New | | | | | | |
|  | Veteran | | |  | Civilian | | |
|  | Beta | t | p |  | Beta | t | p |
| BIAAQ | -.196 | -1.633 | .103 |  | .004 | 0.047 | .963 |
| BICSI | .200 | 2.298 | .022 |  | .189 | 2.783 | .005 |
| PSS | .068 | 0.755 | .450 |  | .065 | 0.901 | .368 |
| Self-compassion | -.210* | -1.653 | .098 |  | -.430 | -5.069 | <.001 |
| EMAS | -.061 | -0.718 | .472 |  | .182 | 2.711 | .007 |
| LOT-R | -.047 | -0.394 | .694 |  | -.184 | -2.330 | .020 |
| MSPSS | .043 | 0.477 | .634 |  | -.027 | -0.440 | .660 |
| PTSD | .136 | 1.152 | .250 |  | .083 | 1.024 | .306 |
|  | SAS-General | | | | | | |
|  | Veteran | | |  | Civilian | | |
|  | Beta | t | p |  | Beta | t | p |
| BIAAQ | -.230* | -1.883 | .060 |  | -.012 | -0.125 | .901 |
| BICSI | .056 | 0.634 | .526 |  | .142 | 2.055 | .040 |
| PSS | .141 | 1.556 | .120 |  | .094 | 1.273 | .203 |
| Self-compassion | -.207 | -1.608 | .108 |  | -.316 | -3.689 | <.001 |
| EMAS | -.087 | -1.004 | .315 |  | .207 | 3.091 | .002 |
| LOT-R | .000 | -0.002 | .998 |  | -.192 | -2.408 | .016 |
| MSPSS | -.076 | -0.837 | .403 |  | -.173 | -2.748 | .006 |
| PTSD | .095 | 0.783 | .428 |  | .154 | 1.829 | .068 |
| * Estimated coefficient statistically significant before imputation but not statistically significant after imputation  PHQ-9: Patient Health Questionnaire (Depression symptoms); SAS-New: Social Anxiety in New Situations; SAS-General: Social Anxiety in General. BIAAQ: Body Image Acceptance and Action (Body Image Psychological Flexibility; BICSI-AF: Body Image Coping Strategies – Appearance Fixing; PSQ: Perceived Stigma; SCS-SF: Self-Compassion; EMAS: Engagement in Meaningful Activities; LOT-R: Optimism; MSPPS: Multidimensional Perceived Social Support; ITQ: International Trauma Questionnaire (PTSD). | | | | | | | |
